# Using Community-Based Participatory Research to Design, Conduct, and Evaluate Randomized Controlled Trials with American Indian Communities

**DOI:** 10.5888/pcd17.200099

**Published:** 2020-11-12

**Authors:** Elizabeth Rink, Kelly Knight, Colter Ellis, Alma McCormick, Paula FireMoon, Suzanne Held, Eliza Webber, Alexandra Adams

**Affiliations:** 1 Department of Health and Human Development, Montana State University, Bozeman, Montana; 2 Department of Sociology and Anthropology, Montana State University, Bozeman, Montana; 3 Messengers for Health, Crow Indian Reservation, Crow Agency, Montana; 4 Fort Peck Community College, Fort Peck Indian Reservation, Poplar, Montana; 5 Center for American Indian and Rural Health Equity, Montana State University, Bozeman, Montana

## Abstract

**Purpose and Objectives:**

Academic literature indicates a need for more integration of Indigenous and colonial research systems in the design, implementation, and evaluation of randomized controlled trials (RCTs) with American Indian communities. In this article, we describe ways to implement RCTs with Tribal Nations using community-based participatory research (CBPR) principles and practices.

**Intervention Approach:**

We used a multiple case study research design to examine how Tribal Nations and researchers collaborated to develop, implement, and evaluate CBPR RCTs.

**Evaluation Methods:**

Discussion questions within existing tribal–academic partnerships were developed to identify the epistemologic, methodologic, and analytic strengths and challenges of 3 case studies.

**Results:**

We identified commonalities that were foundational to the success of CBPR RCTs with Tribal Nations. Long-standing community–researcher relationships were critical to development, implementation, and evaluation of RCTs, although what constituted success in the 3 CBPR RCTs was diverse and dependent on the context of each trial. Respect for the importance of diverse knowledge systems that account for both Indigenous knowledge and colonial science also contributed to the success of the RCTs.

**Implications for Public Health:**

Tribal–academic partnerships using CBPR RCTs must include 1) establishing trusted CBPR partnerships and receiving tribal approval before embarking on RCTs with Tribal Nations; 2) balancing tribal community interests and desires with the colonial scientific rigor of RCTs; and 3) using outcomes that include tribal community concepts of success as well as outcomes found in standard colonial scientific research practices to measure the success of the CBPR RCTs.

**Summary**

**What is known about this topic?**

Research is needed to provide ways to implement randomized controlled trials (RCTs) in Indigenous communities using community-based participatory research (CBPR) practices and principles.

**What is added by this report?**

We used a multiple case study research design to examine how Tribal Nations and researchers collaborated to develop, implement, and evaluate CBPR RCTs.

**What are the implications for public health practice?**

Tribal–academic partnerships using CBPR RCTs must balance tribal community interests with colonial scientific rigor and use outcomes that include tribal community concepts of success as well as outcomes found in standard colonial scientific research practices.

## Introduction

Intervention science highlights the need for the integration of Indigenous-based theories and knowledge systems, community-based participatory research (CBPR) methods, and colonial scientific research strategies in the design, implementation, and evaluation of randomized controlled trials (RCTs) with American Indian (AI) communities (1,2). The Center for American Indian and Rural Health Equity (CAIRHE) at Montana State University in Bozeman, Montana, is tasked with the design, implementation, and evaluation of CBPR RCTs with Tribal Nations. In this article, we assess the implementation of CBPR principles and practices in the following RCTs:

- Nen ŨnkUmbi/EdaHiYedo (“We Are Here Now”): March 2019–September 2023
- Báa nnilah: September 2017–January 2020
- Healthy Children, Strong Families 2: February 2013–April 2017

CAIRHE’s work with Tribal Nations is organized into 3 domains — epistemologic, methodologic, and analytic — to conceptualize the integration of Indigenous and colonial research systems into RCTs. Epistemologic conflicts arise when RCTs, the gold standard for causal interpretation in colonial science (ie, science grounded in the white, predominately Judeo-Christian ideals of the non-Indigenous culture), are prioritized over Indigenous or other knowledge systems (ie, knowledge that comes from local communities) (3–5). Methodologic complexities must be negotiated when conducting RCTs with AI communities (6), because they are frequently geographically isolated, under-resourced, and mistrustful of outside researchers. These issues make engagement, recruitment, and retention in RCTs challenging (7). Furthermore, control groups may be deemed culturally inappropriate by tribal members, given a traditional culture of collectivism that values inclusion and sharing of benefits (3,8–10). Analytic challenges threaten established concepts of validity and reliability in RCTs designed by using colonial science methods (11). For example, RCTs with Indigenous communities may include culturally distinct groups and small populations. These sample characteristics may result in underpowered analyses that call into question external validity and generalizability (12). Thus, analytic strategies that include alternative designs, mixed methods, and statistical computations to maximize power are warranted (13). In addition, RCTs with AI communities may require longer timelines to address tribal norms regarding research sovereignty and ethics (14).

## Purpose and Objectives

Given these issues, we aim to offer insight to support the implementation of RCTs that join social context and human agency with CBPR, Indigenous knowledge systems, and the rigors of RCT designs (15). We discuss our methods and the strengths and challenges of 3 CBPR RCTs in varying implementation stages in culturally distinctive Tribal Nations as examples of how we addressed our epistemologic, methodologic, and analytic differences. More information on the epistemology, methods, and analytics used in our CBPR RCTs is presented in Table 1.

**Table 1:** Project Overview, Epistemology, Methodology, and Analytics Related to Research Design for Randomized Controlled Trials, Case Studies Conducted in 3 US Tribal Nations

Category	Project 1: Nen ŨnkUmbi/EdaHiYedo (“We Are Here Now”)	Project 2: Báa nnilah	Project 3: Healthy Children, Strong Families 2
**Project** **Overview**	• All 15- to 18-year-old tribal member youths living on the Fort Peck Reservation receive intervention using a stepped-wedge design. • Aim is to reduce SRH disparities by increasing birth control use, reducing number of sex partners, delaying onset of sexual intercourse, increasing parent–child communication about SRH, strengthening connections to traditional cultural beliefs, and expanding access to SRH services for youth on the Fort Peck Reservation.	• Members of the Absáalooke (Crow) Tribe aged 25 or older who have chronic illnesses are eligible to participate in the intervention. • In Montana, AIs who have chronic illnesses die earlier than Whites. This program aims to erase this disparity, improve quality of life, increase satisfaction in and participation in social roles and activities, decrease social isolation, increase patient activation, and decrease depression among community members who have chronic illness.	• Intervention included caregivers with 2- to 5-year-old children at 4 reservations and 1 urban Indian health center. • Goal was obesity prevention and healthy lifestyle promotion by improving fruit and vegetable consumption, physical activity, and sleep habits, and by decreasing screen time, stress, and consumption of sugared beverages and junk food.
**Epistemology**	• Strengthening of Assiniboine and Sioux traditional beliefs and practices related to SRH, families, and culture • Integration of Assiniboine and Sioux worldview with Ecological Systems Theory	• Intervention methods co-developed by partners include Crow-specific goal setting, use of traditional stories in intervention, and Crow-based resilience exercise • Participants meet in groups to provide support to one another, strengthening the traditional Crow practice of people coming together and visiting	• Family-based intervention based on the Native concept of elders teaching younger generations • Designed by AI communities in partnership with academic researchers • Based on Social Cognitive Theory and Family Systems Theory
**Methodology**	• Randomized controlled trial with stepped-wedge design • Developed based on previously conducted community focus groups, key information interviews, and a school-based youth survey • Multilevel, multicomponent intervention including “Native Stand” curriculum (individual level), adaptation of “Native Voices” (family level), cultural mentoring program (community level), and adaption of CDC evidence-based best clinical practices of SRH services for youth (systems level)	• All participants receive intervention via wait-list control design • Participants recruited through use of Crow clan system and close kinship	• Randomized controlled trial with modified crossover design • Multicomponent, home-based intervention with 12 monthly mailed lessons for young families • All families received wellness intervention and safety intervention (active control) per community desire for full inclusion • Lessons themed on 6 healthy behaviors or on 11 safety topics; each lesson included AI books, recipes, and games • Intervention supported by Facebook group and biweekly text messages to caregivers • Local site coordinators engaged families in survey and anthropometric data collection every 6 months
**Analytics**	• Mixed-methods approach • Surveys administered to youth and parents to assess components at the individual, family, and community levels • Work plan and quarterly meetings used to assess systems-level component • Focus groups with youth, parents, school personnel, SRH service providers, and mentors held to assess fidelity and acceptability • All data reviewed and interpreted with community advisory board and Fort Peck/MSU-based researchers	• Mixed-methods design to gather contextual data • All qualitative data co-analyzed by community and university partners using co-developed methods • Annual community meetings in reservation districts held to share information and gather input	• Primary comparison will be supplemented by analysis of covariance for BMI/zBMI at Year 1 with randomization and BMI/zBMI at baseline as model terms. • Healthy behaviors and self-efficacy for behavior change from baseline to Year 1 will be analyzed as secondary outcomes. Focus groups were conducted to access feasibility and acceptability of intervention. Qualitative data collaboratively analyzed with communities. • Site-specific baseline data reports were given to each tribe, and aggregated results will be given to each community with time for community input before publication.

Abbreviations: AI, American Indian; BMI, body mass index; CDC, Centers for Disease Control and Prevention; MSU, Montana State University; SRH, sexual and reproductive health; zBMI, BMI *z*-score.

## Intervention Approach

### Project 1: Nen ŨnkUmbi/EdaHiYedo (“We Are Here Now” or N/E)

Sexual and reproductive health (SRH) disparities are higher in AI youth than any other adolescent population in the United States (16–19). Research documents that SRH disparities among AI youth are markers of underlying issues at the individual, family, school, community, and systems levels in AI communities that warrant novel, multifaceted community-based interventions (20–22). N/E is a multilevel, multicomponent, mixed-methods SRH intervention that was developed as a result of a 14-year collaborative research partnership between the Fort Peck Tribes and researchers at Montana State University. The RCT design is based on Fort Peck tribal members’ desire to implement a holistic SRH intervention for AI youth aged 14 to 18. The conceptual framework for N/Es conceptual framework is CBPR, and its theoretical framework is Ecological Systems Theory. A community advisory board of 6 Fort Peck tribal members provided oversight and guidance for the intervention.

N/E includes 1) a school-based SRH curriculum called “Native Stand,” designed to address individual-level factors that lead to sexual risk behaviors; 2) a family-level curriculum called “Native Voices,” tailored to increase communication between adult family members and youth about SRH topics; 3) a cultural mentoring component at the community level that connects AI youth with elders to discuss traditional AI beliefs and practices about SRH; and 4) a systems-level component that works with the Fort Peck epidemiologic team, a multisectoral network of health care providers that delivers SRH services to AI youth to increase coordinate and access to SRH services for AI youth. The systems-level component is based on the Centers for Disease Control and Prevention’s Evidence-Based Clinical Best Practices for Family Planning Services for Adolescents, which for the purposes of N/E was adapted by the Fort Peck epidemiologic team and Montana State University (MSU) researchers for application with the Fort Peck Tribes (23). 

N/E’s school, parent, and cultural mentoring components are evaluated using a cluster-randomized, stepped-wedge design. In the stepped-wedge design for N/E, the 5 schools that Fort Peck AI youth attend are considered clusters that are randomly assigned to the intervention one at a time, with all schools eventually being assigned (24–26). The 5 schools are located in culturally distinct, separate communities throughout the reservation, mitigating the potential for cross-contamination. Our selection of a stepped-wedge design to evaluate N/E is consistent with tribal members’ desires that all adolescents aged 14 to 18 years receive the intervention, as well as providing the opportunity for comparisons between school clusters that have received N/E and those that have not. The systems-level component of N/E is measured qualitatively, and N/E has completed its first year of intervention implementation in one of the schools on the Fort Peck Reservation.

### Project 2: Báa nnilah

The Apsáalooke (Crow) language phrase Báa nnilah, translates to “advice or instructions for life received from others” in English. The Báa nnilah program is a chronic illness self-management study funded by an R01 grant from the National Institute on Minority Health and Health Disparities (NIMHD) that tests the effects of the intervention versus usual care by using a wait-list control group design. It was conducted among 211 randomly assigned AI men and women aged 25 years or older who have chronic illness and live on the Apsáalooke Reservation. To mitigate contamination, participants were assigned to intervention and control groups by using cluster randomization, where participants could select to be randomized with others who were in their household or who were closely related. In Montana, AI individuals die 14 years earlier from heart disease than people who are not AI, 11 years for those with diabetes, and 12 years for those with cerebrovascular disease (27). Despite their potential to lower mortality rates and improve quality of life, existing programs that address chronic illness management fail because they are not consonant with AI culture (28).

### Project 3: Healthy Children, Strong Families 2 (HCSF2)

Obesity rates in AI children are among the highest of all races and ethnicities, and obesity greatly increases the risk of later chronic disease (29). HCSF2 was an NHLBI R01-funded RCT of a healthy lifestyle intervention for AI families with children aged 2 to 5 years. HCSF2 targeted multiple healthy lifestyle and obesity prevention goals; the methods are described in detail elsewhere (30). The HCSF2 design was based on an initial, smaller RCT intervention designed with 4 Wisconsin communities, and after achieving positive results it was expanded to the larger national RCT with 5 AI communities (30). In HCSF2, all 5 participating communities did not want to have a passive control group, so a modified crossover design was chosen with an active control intervention that focused on safety. Safety was chosen because accidents and injuries are the leading cause of illness and death in AI children in this age group, and it was proposed by one of our long-standing partner communities as being a good control group (31). 

Families with young children from 5 AI communities nationwide were randomly assigned to a child safety control group (“Safety Journey”) or to a healthy lifestyle intervention (“Wellness Journey”) for 1 year. Members of the latter group were mailed a monthly intervention toolkit that focused on a healthy lifestyle, augmented with social support through Facebook and text messaging. Families in the child safety control group received monthly safety newsletters, with additional mailed books and supplies. All families randomly assigned to the intervention group in year 1 received the control intervention in year 2, and families randomly assigned to the control group in year 1 received the wellness intervention in year 2. Families were assigned to start either in the Wellness Journey or the Safety Journey and stratified by tribal site and child weight status (overweight vs healthy weight). Random assignment was conducted by the REDCap data management system (Vanderbilt University) using a permuted block strategy. Between-group differences in primary outcomes (adult body mass index [BMI] and child BMI *z*-score) and secondary outcomes (health behaviors) were assessed after year 1, which functioned as a traditional RCT, and again after year 2 (30).

The RCTs met CONSORT guidelines for clinical trials (32). All 3 of our RCTs were conducted within a CBPR framework, having been developed with extensive tribal support and tribal–academic partnerships ranging from 5 to 20 years. The 3 RCTs also had community advisory boards that provided oversight, direction, and cultural knowledge for the studies. The incorporation of colonial science RCT designs, CBPR, and expertise based on tribal culture in the 3 studies informed the integration of Indigenous ways of addressing health disparities and colonially defined, scientifically rigorous research with Tribal Nations (1). 

Ethical approval for the RCTs depended on the ethical approval process required by the Tribal Nations in which the RCTs took place. In some instances, Tribal Nations had an established institutional review board (IRB), which provided ethical approval for the studies. In other instances, there was no established tribal IRB and the project was approved by the tribal council. The tribal ethical approvals obtained for our RCTs underscore the importance of Tribal Nations’ research governance authority, their role as stewards and keepers of research conduct on their lands and with their people, and their rights to make decisions and control the research they are involved with (33,34). Finally, the RCTs received approval from the IRB at MSU (Projects 1 and 2) or the University of Wisconsin School of Medicine and Public Health (Project 3).

## Evaluation Methods

We used a multiple case study research design to examine the ways in which Tribal Nations and CAIRHE researchers collaborated using CBPR to develop and implement RCTs (35,36). Several steps were taken to facilitate this process. First, CAIRHE researchers met to discuss implementing RCTs in which, at the request of our tribal partners, all eligible tribal members in the study population would be included in the intervention, thereby negating the role of a control group. This conversation led to a broader discussion on how CBPR principles and practices are used to assess, negotiate, and determine the most effective intervention strategy that honors tribal interests, needs, and Indigenous worldviews while maintaining the integrity of the research protocols inherent in conducting RCTs. Second, on the basis of this discussion, we generated a list of questions to discuss with our community partners about the process undertaken by each of the 3 tribal–academic partnerships to arrive at their chosen RCT design (Box). Third, community partners and CAIRHE researchers met to discuss the list of questions and identify the pertinent issues that went into selecting our respective RCTs. Fourth, CAIRHE researchers reviewed and discussed the main themes that emerged from the conversations with our community partners. We identified 3 research design concepts — epistemology, methodology, and analytics — that are central to understanding and determining the integration of CBPR with the development and implementation of an RCT in Tribal Nations. Finally, community partners and CAIRHE researchers finalized the main research design concepts and determined the strengths and weaknesses of their respective RCTs.

**Box. Community-Based Participatory Research and Randomized Controlled Trial (RCT) Development Discussion Questions for Community Partners and Center for American Indian and Rural Health Equity Researchers**

• What is your research project?

• What ethical concerns motivated your research design and how did you identify them?

• What obstacles did/do you face in setting up an RCT?

• What conversations did you have with the community (community stakeholders or your community advisory board)?

• At what stage of the partnership were you ready to implement an RCT?

• What statistical methods did you use?

• What were the pros and cons of how you ultimately designed the RCT?

• Which RCT did you want and which did you get?

• What lessons learned and suggestions/tips do you have for researchers approaching an RCT in a tribal community for the first time?

## Results

We present our results starting with a newly established CBPR RCT and closing with a completed CBPR RCT. The studies varied in size and scale from tribally based (Báa nnilah, N = 211 and N/E, N = 451) to nationally based (HCSF2, N = 450 child–caregiver dyads), with each targeting different age groups: children (aged 2–5 years and their caregivers), adolescents (aged 14–18 years), and adults (aged 24–82 years) (Table 2). Collectively, our findings reflect a diverse range of CBPR RCT study designs in Tribal Nations. For each study, we show the strengths and challenges identified in the research design.

**Table 2:** Participant Demographics, Case Studies of Implementing Randomized Controlled Trials in 3 US Tribal Nations

Demographic Characteristic	Nen ŨnkUmbi/EdaHiYedo (“We Are Here Now”) (N = 456)	Báa nnilah (N = 211)	Healthy Children Strong Families 2 (N = 450 Families)
**Age**	14–18 years	24–82 years	Children aged 2–5 years and their adult caregiver
**Sex, no. (%)**			
Male	227 (50)	59 (28)	Caregiver: 24 (5.3)/child: 224 (49.8)
Female	222 (49)	152 (72)	Caregiver: 426 (94.7)/child: 226 (50.2)
Neither male nor female	5 (1)	NA	NA
**Location**	Fort Peck Reservation	Crow Reservation	4 tribal reservations (1 in the Northeast, 2 in upper Midwest, and 1 in the Northern Mountain region) and 1 urban clinic serving a primarily American Indian population in the Southwest

Abbreviation: NA, not applicable.

### Project 1: Nen ŨnkUmbi/EdaHiYedo (“We Are Here Now,” or N/E)

**Strengths.** N/E is grounded in a long-standing research partnership between the Fort Peck Tribes and MSU researchers. N/E is modeled after a holistic, inclusionary Assiniboine and Sioux worldview that honors the individual, the importance of family, the need to instill and revitalize cultural beliefs and values in tribal youth, and the awareness that large and complex systems such as schools and health care services influence the SRH of young people. N/E’s other strengths are the adaptation and efficacy testing of 2 previously developed curricula for tribal youth and families, the integration of an existing tribal cultural mentoring program, and the enhancement of SRH services for AI youth following evidenced-based clinical practices for adolescent family planning services.

**Challenges.** Challenges to N/E include 1) the use of self-reported measures of sexual risk behaviors to assess our primary, secondary, and tertiary outcomes; 2) social desirability bias because of research participants’ familiarity with the community members implementing N/E; 3) the potential of recruitment and retention of youth and families to affect sample size and statistical power; 4) the possibility of contamination to sites that have not yet entered or completed the intervention; and 5) the possibility of an unobserved 3-way interaction existing by intervention cluster under the stepped-wedge design.

### Project 2: Báa nnilah

**Strengths.** Báa nnilah originated in the community and uses trusted community members to facilitate it. Similar to the other RCTs, we are building on a long-term partnership. Intervention methods are centered on Apsáalooke cultural strengths, and content is based on our conceptual framework of influencers of self-care related to chronic illness that came from community interviews.

**Challenges.** Community members in the intervention group shared information with wait-list control group members. Although contamination is a problem during data analysis in an RCT, sharing information between groups is seen, from the community’s perceptive, as a strength and is in keeping with the Apsáalooke cultural value of inclusiveness. We gathered data on contamination to assess the impact of sharing. Similar to the other 2 projects, the ability for participants to attend all of the program gatherings was affected by inclement weather, lack of transportation, travel conflicts, and deaths in the community.

### Project 3: Healthy Children, Strong Families 2 (HCSF2)

**Strengths**. This multisite, 5-state trial had a strongly inclusive RCT design that enabled us to have 100% recruitment and excellent family retention in the study, with loss of only 16% over 2 years (30). The safety active control group was well received, as it allowed for no passive control communities and no wait lists for families, which enhanced family recruitment and reduced attrition. The AI value of inclusivity was used in teaching components appropriate for both adults and children in the family, and these components incorporated culturally appropriate books, recipes, and activities. Each community had a local HCSF2 community coordinator who was responsible for helping families with survey completion, doing anthropometric measures, and creating local Facebook content. These local coordinators were critical to keeping families connected and reducing attrition. Sites with stable local coordinators were able to meet or exceed recruitment targets. A central university site coordinated the study and distributed all mailed toolkit materials, reducing the burden for individual sites. All families had interest in and engagement with the study materials, and focus groups described substantial benefits to study participation. 

**Challenges**. Conducting a multisite, randomized trial using community-engaged approaches requires flexibility. Geographic dispersion of the 5 communities in 3 time zones necessitated significant planning and made it impossible to use more robust outcome measures such as accelerometry. In addition, it was important to engage in discussions with more potential sites than needed because of dropout during the early stages of project planning. Every site presented a different challenge. Flexibility with local hiring and staff turnover, site timing, and recruitment were necessary. In addition, because of the community value for inclusivity and wish not to stigmatize children, we had to stratify the randomization to include equal numbers of children above and below the 85% BMI in each group, thus reducing our ability to show change in BMI. However, this stratification improved recruitment and emphasized the importance of prevention. Lastly, because all families viewed themselves as being in the HCSF2 study, some families may have started to make healthy lifestyle changes during the first year despite being in the control group.

## Implications for Public Health

Respective tribal–academic partnerships demonstrated that long-term CBPR collaborations can mitigate the epistemologic, methodologic, and analytic complexities of conducting RCTs with AI communities (37–40). CBPR provides a set of principles and guidelines that help communities and researchers build equitable, mutually beneficial, and long-term partnerships to create locally applicable knowledge (41). AI communities and research partners may, however, approach these collaborations from different perspectives to design and implement studies that fit community priorities and satisfy salient cultural contexts and specific RCT conventions (42). Recognizing the variation in the types of CBPR RCTs presented here, we identified some commonalities as cornerstones of successful CBPR RCTs (Table 3). By so doing, we generated new knowledge that supports efforts to decolonize research methods with Indigenous peoples and develop new gold standards for RCTs with Tribal Nations (2).

**Table 3:** Using Community-Based Participatory Research in Randomized Controlled Trials, Common Themes in Case Studies Conducted in 3 US Tribal Nations

Theme	Elements
Long-standing partnerships	• 5- to 20-year partnerships • Open dialogue on the most appropriate RCT to meet tribal interests • Strength of partnership to withstand the test of time and changes in tribal leadership and personnel • Co-learning and sharing between tribal members and researchers • Capacity to find solutions to meet tribal needs and the rigor of RCT designs

Diverse concepts of success	• Substantial community engagement before the design and implementation of an RCT can increase its success • Hiring tribal members to work on implementation and data collection enhances the success of the RCT • RCT research participants’ sharing of information is in accordance with tribal cultural philosophies of inclusiveness • Identify an RCT design that ensures all eligible tribal members have the opportunity to participate in the intervention

Respect for diverse knowledge systems	• Understanding and integrating traditional Indigenous knowledge systems and ways Indigenous people view the world with knowledge based on a colonial worldview • Marrying CBPR principles and practices with Indigenous cultural beliefs and practices and the rigors of RCT standards and requirements • Layering of traditional knowledge and colonial science knowledge with contemporary culture on reservations, which is neither traditional nor modern • Tribal ethical approval as crucial component of tribal sovereignty and Tribes rights to make decisions about what kind of research and how research is conducted on their lands and with their peoples

Abbreviations: CBPR, community-based participatory research; RCT, randomized controlled trial.

First, long-standing community–researcher relationships were identified as foundational to the development, implementation, and evaluation of CBPR RCTs. All partnerships presented had 5 to 20 years of continuous successful engagement to establish trust and build capacity. The success of our respective RCTs was based on the substantial amount of community engagement *before* the RCTs’ design and implementation, which allowed the researchers to develop an understanding of cultural beliefs and practices and tribal community dynamics and processes. Multiple studies conducted in AI communities have confirmed the importance of tribal support and trusting relationships to ensure project success (43,44). However, we conclude that the established depth of trust in partnerships developed over multiple years fostered an open dialogue between community members and researchers that led to the strengths in our respective studies, including 1) integrating the best-suited RCT design with the cultural beliefs and practices, structural systems, and health needs of the community; 2) the ability of our partnerships to withstand the test of time and changes in tribal leadership; 3) exchange of Indigenous knowledge and colonial science to support co-learning between community members and researchers; and 4) the capacity to find solutions to challenges that met the cultural, structural, and health needs of the community in addition to the rigor of RCT designs. 

Second, in our CBPR RCTs, the values and definition of success differed between the communities and researchers. For example, there was the potential for Báa nnilah participants in the intervention group to share information with wait-list control group members. From a purely colonial science research perspective, this type of “contamination” compromises the validity of the study’s results. From a community perspective, sharing information among groups is culturally appropriate and in harmony with an Indigenous worldview of collectivism. However, other concepts of validity warrant exploration here. In CBPR, validity of a study can be based on such attributes as the characteristics of the partnership in which a study is conducted and the process the partnership went through to design, implement, and evaluate a study (39,45). Thus, future tribal–academic partnerships that implement RCTs may consider how to establish validity within the context of their research.

Similarly, inherent in RCTs is the need for some form of control group or groups in which a portion of a population either does not receive the intervention or receives a placebo. From the perspective of the communities involved in these RCTs, the idea that a person or group of people would not receive something that other people receive, thereby dividing people into those that have and those that do not have, is culturally unacceptable. As academic partners, we found ourselves needing to identify RCT designs that ensured that all tribal members who were eligible for the study would be able to participate. 

Our final differing area of success is related to social desirability. In terms of research, social desirability is viewed as a limitation that negatively affects research results because it suggests that participants will respond in a certain way if they know the members of the research team who are collecting the data. In contrast, tribal members will participate in research studies specifically because they know and feel comfortable with members of the research team, thereby enhancing not only the depth of responses given but also the recruitment and retention of participants needed in RCTs.

Our examples of the difference between the scientific needs of the researchers and cultural needs of the Tribal Nations in which our interventions are implemented underscores emergent intervention science research. Our 3 RCTs highlight current intervention science research with Tribal Nations that supports culture as an organizing factor in how RCTs are designed, implemented, and evaluated with Indigenous communities (46). We recommend researchers working with Tribal Nations to design, implement, and evaluate RCTs identify and prepare for how to address diverse approaches and perspectives to research in their work together to generate new knowledge related to intervention science with Tribal Nations.

Third, the path toward our respective CBPR RCTs generated a common respect for the importance of diverse knowledge systems. Dickerson et al illustrate strategies for the integration of culturally centered interventions and colonial-based research practices and theories with AI communities (2). Their work outlined techniques such as including talking circles, community partnership committees, and tribal leader and elder knowledge and expertise to adapt non-Indigenous research strategies such as focus groups, surveys, and interviewing. In our experiences, community members and university researchers shared multiple conversations over several years in which we grappled with integrating Indigenous wisdom, traditional knowledge systems and ways of viewing the world, and colonial science knowledge systems. Furthermore, today there is a contemporary culture on reservations that is neither traditional nor modern but rather a merging of the two. This reality underpins how things actually happen on reservations, which supports the importance of evaluating how an intervention was implemented versus what was planned for intervention implementation (37,45). Contemporary reservation culture creates yet another form of knowledge to be understood and integrated into the design, implementation, and assessment of RCTs with Tribal Nations. This multifold process of sifting through Indigenous wisdom, traditional knowledge systems, contemporary reservation culture, and colonial scientific methods required reflection and critique of established RCT methods to create new standards for measuring the attainment of our CBPR RCT aims. 

It is critical for those working in designing clinical trials and for funders to understand that in underserved communities, colonial definitions of trial success with primary, secondary, and tertiary outcomes that are strictly defined and measured may not capture the real success of an RCT in a tribal community. For example, although HCSF2 did not show a difference in the primary outcome of BMI *z*-score change in children, family engagement with the project was substantial and led to lifestyle improvements and readiness to change, as well as the unanticipated positive outcome of families spending more time together. Several Tribal Nations also experienced an increased interest and commitment to promoting child and family health, thereby likely contributing to long-term health improvements in their communities. Engaging as a community in a health improvement trial in itself can be beneficial for that community, and this success is not necessarily dependent on the scientific definition of success for the project.

Our study has limitations. The interventions presented are in different stages of implementation. In addition, the interventions address different age groups and health disparities, and they take place in culturally and socioecologically diverse Tribal Nations in Montana and other areas of the United States. The interventions also use 3 different RCT designs with CBPR being the foundational framework for each study. These distinct variations in our respective interventions may make it difficult to generalize our identified epistemologic, methodologic, and analytic issues to other tribal settings. However, we were able to identify common cornerstones for successful CBPR RCTs with AI communities even with our interventions’ apparent contrasts. Our identified conclusions can serve as recommendations for future CBPR intervention science practices with Indigenous groups.

We have presented the epistemologic, methodologic, and analytic issues inherent in the design, implementation, and evaluation of CBPR RCTs with Tribal Nations and the strengths and challenges that our differing CBPR RCTs encountered. On the basis of our collective experiences, we believe that future CBPR RCT tribal–academic partnerships must understand several key points. First, it is critical to take the time (ie, several years) to establish trusted, meaningful CBPR partnerships before embarking on RCTs with Tribal Nations. Although extensive community engagement before conducting an RCT with a tribal community may seem unrealistic from an academic perspective, strong relationships between tribal members and researchers are foundational to working through the complexities of RCT research. Second, there is a need to balance community interests and desires with expectations established by funders, such as the National Institutes of Health (NIH), to meet the scientific rigor of an RCT. The balance between community needs, colonial-focused research precision, and funding expectations can be sorted out through a combination of iterative communication between tribal members, researchers, and funders, as well as promoting co-learning with tribal members, researchers, and policy makers in funding agencies that support the science of RCTs with Indigenous communities. Third and finally, success in CBPR RCTs must be measured using both tribal community concepts of success and positive outcomes and outcomes found in standard colonial scientific research practices. This broader definition of success will more significantly benefit the communities involved and may lead to improved intervention recruitment and sustainability.
